# ERP Biomarkers of Auditory–Visual Distraction in Aging and Cognitive Impairment

**DOI:** 10.3390/brainsci15111242

**Published:** 2025-11-19

**Authors:** Valentina Gumenyuk, Oleg Korzyukov, Sheridan M. Parker, Daniel L. Murman, Nicholas R. Miller, Matthew Rizzo

**Affiliations:** Department of Neurological Sciences, University of Nebraska Medical Center, Omaha, NE 68131, USA; okorzyukov@unmc.edu (O.K.); sheparker@nebraskamed.com (S.M.P.); dlmurman@unmc.edu (D.L.M.); matthew.rizzo@unmc.edu (M.R.)

**Keywords:** distraction, event-related potentials, aging, Alzheimer’s disease, and mild cognitive impairment

## Abstract

**Background/Objectives**: Distraction is a form of impaired selective attention that becomes more pronounced with normal aging and in pathological conditions such as mild cognitive impairment (MCI) and Alzheimer’s disease (AD). Event-related potentials (ERPs) provide sensitive, time-resolved measures of neural mechanisms underlying distractibility. This study aimed to identify age- and disease-related ERP signatures of auditory–visual distraction as potential functional biomarkers for cognitive decline. **Methods**: Forty-six participants were enrolled, including young controls (Y), healthy older controls (O), individuals with MCI, and individuals with AD. Participants performed cross-modal interference tasks in which irrelevant auditory distracting sounds were paired with a relevant visual discriminating task. The distraction potential was quantified as the difference between ERP responses to novel distractors and standard stimuli, focusing on three core components: N1-enhancement, P3a, and reorienting negativity (RON). Behavioral measures (accuracy, reaction time, miss responses) were also assessed. **Results**: Compared to Y, O showed increased N1-enhancement and reduced P3a and RON amplitudes, consistent with age-related susceptibility to distraction. Patients with MCI and AD exhibited further abnormalities, including diminished P3a and altered RON responses, suggesting impaired orientation and reorientation of attention. Behavioral distraction effect was observed in all groups, with no significant difference between groups. ERP–cognition correlations indicated that reduced P3a amplitude and delayed RON were associated with executive dysfunction and memory deficits. **Conclusions**: ERP signatures of distraction, particularly altered P3a and RON components, differentiate normal aging from pathological decline and may serve as functional biomarkers for early detection of MCI and AD. These findings highlight the translational potential of distraction paradigms in clinical assessment of aging-related cognitive impairment.

## 1. Introduction

From a very young age, humans begin to develop selective attention—the ability to ignore irrelevant information and focus on stimuli that are relevant and important to the task at hand. Within this domain, distraction refers to the process by which task-irrelevant stimuli capture attention and interfere with ongoing performance. Distraction can be identified based on two criteria: (1) evidence that the distracting stimuli have been processed, and (2) evidence that performance on the primary task has been impaired as a result [1].

Like other brain functions, distractibility follows a developmental trajectory [2,3] and an aging course [4]. It can also be influenced by attention disorders [5,6]. Age-related changes within the normal range may be driven by declines in hearing and/or vision leading to reduced processing of acoustic or visual stimuli [7]. However, when aging follows a healthy course, the cognitive functions that support selective attention may be preserved [8]. In such cases, the use of hearing aids and/or vision correction could help maintain performance by supporting the ability to focus on relevant information while filtering out extraneous stimuli [9]. However, a meta-analysis by Loughrey et al. [10] reported significant associations between age-related hearing loss and accelerated multidomain cognitive decline including abnormal selective attention function, general cognitive impairment, and dementia specifically. The identification of objective, age and disease-specific biomarkers is critical and a time-sensitive task, as the global incidence of dementia and cognitive impairment continues to rise. Approximately one new case is diagnosed every three seconds worldwide [11]. The research on the effects of aging on auditory processing and auditory cognition, particularly auditory distraction, is in line with that demand for identifying early biomarkers of dementia and cognitive impairment in older people.

The neuronal mechanism of distraction is quite complex and multiphasic that is accurately measured by event-related brain potentials (ERPs), a noninvasive method with accurate time resolution. ERPs, as well as Magnetoencephalography (MEG), permit the study of the subsequent neuronal processes caused by the sound distractor and collectively referred to as the “distraction potential” [12,13].

Electrophysiologically, the distraction potential is characterized by three distinctive event-related brain potentials [2,13]. The first is the N1-enhancement, which reflects the process of change-detection, occurring at a latency of 100–150 ms from the sound onset. The second is the P3a, which reflects the involuntary orientation of attention to the distracting stimulus, occurring at latency of 200–350 ms. Finally, the reorienting negativity (RON) [14], which reflects the reorientation of attention back to the main task after the momentary distraction, occurs at a latency of 400–600 ms after the onset of the distracting sound. Each of these neuronal processes represents the timing and path of processing distracting auditory information in the brain and, at the functional level, underline how dynamic shifts in attention occur when triggered by a distracting sound.

To our knowledge, only a limited number of studies have employed a cross-modal approach to examine age-related differences in susceptibility to auditory distraction, as indexed by behavioral performance during a primary visual discrimination task. Reference [15] reported no significant differences in reaction times (RTs) to visual stimuli between distracting and non-distracting sound conditions. However, their ERP findings revealed age-related differences in the N1 and MMN components reflecting the early change-detection phase of distraction processing. In contrast, Andrés et al. [16] observed a larger behavioral distraction effect in older adults (mean age 67.9 ± 8.8 years) compared to younger adults (mean age 22.2 ± 3.7 years), as reflected by increased RTs. More recently, Schils et al. [17] demonstrated that older healthy participants (64–80 years) exhibited longer RTs (811 ms) in a cross-modal visual–auditory attention-switching task compared to younger adults (19–20 years, RT = 511 ms).

At the behavioral level, distraction is usually observed as deterioration of performance in the current task caused by stimuli extraneous to the task [13,18]. Overall, these behavioral studies on the effects of normal aging on auditory distraction have yielded mixed results. Nevertheless, several investigations converge in showing that older adults are more susceptible to higher distraction, as evidenced by increased RTs, relative to younger and middle-aged healthy individuals [19].

At the brain level, normal aging is associated with structural and functional decline (considered within the normal range)—in the dorsolateral prefrontal cortex, inferior temporal cortex, inferior parietal lobule, with minimal changes in the primary visual cortex [20]. Notably, all of these regions play critical role in filtering task-irrelevant information [21] which is the core neurocognitive process underlying cross-modal distraction. Such neuronal deterioration within specific brain regions is thought to underscore the slowing of inhibitory processes during distraction in healthy aging and may contribute to even more pronounced impairments in cross-modal inhibition control in patients with AD and MCI. Taken together, these findings suggest that age-related deficits in the ability to ignore auditory distraction in cross-modal task are particularly evident in individuals with AD [22] and perhaps may also emerge in patient with MCI, whose brains may be somehow impacted by pathology specifically in temporal—frontal network, leading to higher distractibility. Therefore, studying auditory–visual distraction in AD and MCI has the potential to reveal early neurophysiological alterations that are not detectable through standard global clinical assessments. This approach may provide a sensitive index of early dysfunction within attentional auditory networks, which are the most impacted even by healthy aging, and can serve as a complementary tool for characterizing specific-disease progression.

Both the stated above empirical evidence and everyday experience indicate that aging influences higher distractibility in cross-modal situations. However, the neural mechanisms underlying abnormal distractibility in healthy versus pathological aging remain poorly understood. It is also unclear whether distinct neuropathological processes in AD and MCI give rise to differential neuronal correlates of distraction potential elicited by auditory distracting sounds, and whether such correlates are specific to each disorder. To address this gap, the present study investigated the effects of age and pathology (AD and MCI) on the different phases of the distraction potential elicited by a well-characterized auditory–visual distraction paradigm. Specifically, we examined auditory and visual obligatory evoked potentials as indices of sensory processing across participants, regardless of age or diagnostic status, and further analyzed the phases of the distraction potential as a frontal- temporal lobes probe in normal and pathological aging.

We hypothesized that, despite similarities in obligatory auditory (N100-aud) and visual (N1-vis) responses, aging and pathology (MCI and AD) would differentially affect the involuntary shift in attention. Specifically, in patients with Alzheimer’s disease, the orienting phase of attention (P3a component)—representing the second phase of the distraction potential—was expected to show greater alterations than in patients with MCI, reflecting more pronounced frontal structural degeneration. In addition, we anticipated age-related reductions in the amplitude of the distraction potential between older and younger healthy participants, reflecting the normal aging continuum of attentional control during auditory distraction while performing the visual discrimination task.

## 2. Materials and Methods

### 2.1. Participants

Sixty-four people initially responded to flyers, word-of-mouth advertisements, and/or Memory Disorders Clinic provider invite. Exclusion criteria included major depression, primary sleep–wake disorders, significant hearing loss, severe vision deficit, and use of medications affecting sleep. Based on our criteria, fifty-three individuals aged 25 and older qualified for the study. All interested participants were pre-screened for eligibility in a brief 5–10 min phone conversation.

Forty-six participants passed the phone screening and were invited to provide written informed consent. For participants with pathological aging who might experience cognitive impairments, an additional consent form was provided to and signed by a legal guardian (a family member or close friend) who assisted with daily sleep–wake evaluations through subjective self-reports. The research coordinator-maintained contact with both the participant and their legal guardian, providing reminders via phone calls to ensure accurate recording of the sleep diary and consistent use of the actiwatch throughout the study. The results of objective and subjective sleep data were published elsewhere [23].

After providing informed consent, participants underwent comprehensive cognitive assessments including the Montreal Cognitive Assessment (MoCA), and the version 3 of the Alzheimer Disease Centers’ Neuropsychological Test Battery in the Uniform Data Set (UDSv3, [24]) and a clinical neurological examination was conducted by an experienced neurologist (DLM) to rule out clinical signs or symptoms of stroke or parkinsonism using the Unified Parkinson’s Disease Rating Scale (UPDRS) part 3 motor score.

### 2.2. Neuropsychological Cognitive Assessment

The UDS v3 neuropsychological test battery was subdivided into five domains. Specifically, Memory (Benson Complex Figure Recall, Craft Story), Visuospatial (Benson Complex Figure Copy), Language (category fluency, MINT), Executive Function (Trail Making Test Part B, letter fluency) and Attention (Number span, Trail Making Test Part A). Evidence of cognitive impairment in one of these five domains was met if either the Jak criteria [25] or the Peterson criteria [26] were met for that domain. To avoid potential confounding effects from demographic variables such as age, sex, and education in cognitive assessments, all cognitive evaluation results were normalized using the UDS-3 calculator [27] (see Table 1, *z*-scores).

### 2.3. Classification of Participants

The healthy control cohort did not show signs of cognitive impairment on the neuropsychological test battery and was divided into two subgroups based upon age (young and older, see Table 1). The cognitive impaired groups (based on their cognitive clinical evaluations results) were divided into MCI or dementia based upon the severity of cognitive impairment and level of functional impairment determined with the neuropsychological test battery, Quick Dementia Rating Scale [28], and the Functional Activity Questionnaire [29]. The classification of MCI or dementia was consistent with the syndromic staging guidelines in the 2018 NIA-AA Research Framework [30]. Participants with dementia included in this report all met criteria for probable Alzheimer’s disease using the 2011 NIA-AA diagnostic guidelines for the clinical diagnosis of probable AD dementia [31]. Four AD subjects had spinal fluid AD biomarker confirmation of AD pathology, while the six other AD subjects were diagnosed clinically and met criteria for probable AD, as determined by an experienced neurologist (DLM).

The final sample consisted of 45 participants divided into four subgroups: Young age group (Y, 38.5 ± 8.2 years, n = 12, 6 females), Older age group (O, 68.4 ± 7.7 years, n = 11, 6 females), MCI group (70.1 ± 7.4 years, n = 12, 5 females) and the AD group (69.0 ± 8.5 years, n = 10, 5 females). In the healthy older group, the ERP data were heavily contaminated by frequent blinking artifacts.

All participants were right-handed with normal or corrected to normal hearing and vision and free of psychiatric conditions (see Table 1 for detailed demographic and general characteristics of participants). To control alertness and daytime sleepiness all participants completed Epworth Sleepiness Scale and Insomnia Severity Scale.

The study was approved by the Institutional Review Board (IRB #0887-21-EP, approval date: 21 January 2022) at the University of Nebraska Medical Center, Omaha, Nebraska, USA.

#### Auditory-Visual Distraction Task and Assessment of Distraction Potential

**Figure 1:**
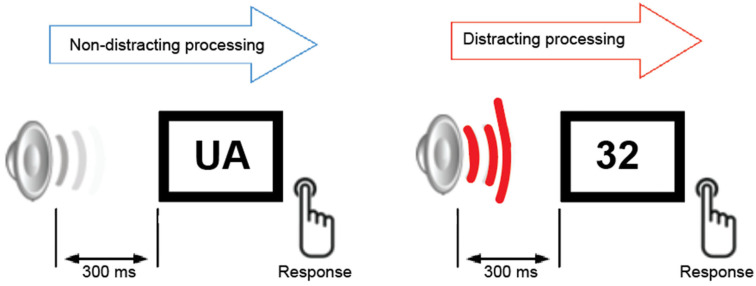
*Auditory distraction–visual discrimination task*. The paradigm included non-distracting (simple tone) and distracting (novel sound) auditory stimuli presented prior to a visual discrimination task (letters vs. numbers). Auditory and visual stimuli were randomized throughout the study. Participants were instructed to ignore the auditory stimuli and focus on discriminating between visual stimuli by pressing the corresponding button in response to letters or numbers.

During the auditory–visual task (Figure 1), all participants were presented with two categories of equiprobable visual stimuli. The visual stimuli were white capital letters and digits (same font and size) presented randomly against a black background at the center of a computer monitor for 200 ms. The viewing distance was approximately 50 cm. Participants were instructed to fixate on the center of the screen and press the left button for letters and the right button for digits on a button response controller. They were instructed to ignore all auditory stimuli and to minimize blinking and body movements. Each visual stimulus was preceded by a task-irrelevant auditory stimulus that was either a pure tone (serving as a non-distracting event 800 Hz, 200 ms duration, probability = 0.8) or a novel sound (800 Hz, 200 ms duration, probability = 0.2; serving as a distracting event). The onset-to-onset interval between auditory and visual stimuli was 300 ms. Each trial included 70 novel sounds drawn from a library of 200 to maintain stimulus novelty, ensuring that each sound was used only once per trial. Prior to the study, all participants underwent hearing threshold screening using a clinical audiometer (frequency range: 250–1000 Hz). Based on the results of the hearing test, no participants were excluded from the study. Ten participants—4 in the older control group, 2 in the MCI group, and 4 in the AD group, regularly used hearing aids in daily life; however, the devices were removed during both the hearing screening and the experimental sessions.

Prior to the experiment, each participant completed a brief (<3 min) training session consisting of a short practice block with visual stimuli only. Participants verbally indicated when they felt comfortable and familiar with the task. The study consisted of three experimental blocks, each containing 380 sound–visual pairs and lasting approximately five minutes.

Participants performed the task while lying comfortably on a study bed inside a sound-attenuated, magnetically shielded room. EEG and magnetoencephalographic data were recorded simultaneously. The MEG findings will be reported elsewhere separately.

### 2.4. ERP Recording

EEG was recorded using a 64-channel EEG cap (10–20 system, Easy Cap, Gilching, Germany) and MEGin system (MEGIN Elekta Neuromag TRUIX, Finland, Helsinki). In addition, electrooculogram (EOG) was recorded using two electrodes attached to the left and right canthus of each eye, and two electrodes attached below and above the left eye. All impedances were kept <10 kΩ during each session. A band-pass filter was set from 0.1 to 300 Hz, and the sampling rate was 1000 Hz. EEG data were analyzed using Brain Vision Analyzer software version 2 (Brain Products GmbH, Gilching, Germany). ERP data were segmented separately for distracting and non-distracting stimuli, and each segment was started 100 ms prior to stimulus onset and continued for 800 ms after the stimulus onset. The first five trials were excluded from the offline analysis to minimize potential novelty effects associated with the initial auditory stimuli. A bandpass filter ranging from 0.1 to 40 Hz was applied to segmented data. Segments in which the EEG exceeded ±100 μV were excluded from the average. ERPs in response to distracting and nondistracting stimuli were averaged separately. On average, at least 90 distracting and 140 nondistracting stimuli were included for each subject’s grand average for each session. Baseline correction (100 ms prestimulus interval) was applied to the averaged data.

### 2.5. Behavioral Analysis of Distracting Potential

A correct response within a 1000 ms interval after visual stimulus onset was considered as “correct”. An incorrect button press during this interval was classified as an “error,” and a response beyond the 1000 ms interval or no response was classified as a “miss.” Mean response time (RT), correct, error, and miss rates were calculated separately for distracting and nondistracting stimuli. Mean RT and behavioral rates were calculated using MATLAB R2024a (MathWorks, Natick, MA, USA).

For each participant, the obligatory auditory N1 was defined as the largest negative deflection within the 80–120 ms latency window. N1 peak amplitude was measured at the FCz electrode in ERPs elicited by both distracting and non-distracting sounds. The distraction potential was computed as a difference waveform by subtracting ERPs to non-distracting trials from those elicited by distracting trials.

Grand-averaged difference waveforms were then used to identify distraction-related brain responses, including N1 enhancement, P3a, and RON. The mean amplitude of N1 enhancement was quantified within the 130–180 ms latency window at the FCz electrode. The P3a is a biphasic brain response, consisting of early (e) and late (l) phases, previously reported in children and young adults [32]. In the present study, the late P3a—associated with involuntary orienting to novelty—was examined as the mean amplitude within the 250–370 ms latency window for each participant. Finally, the mean amplitude of the RON component was measured within the 420–550 ms latency window. All time windows were determined based on the grand average waveforms for each group.

### 2.6. Statistical Analyses

*Behavioral distraction effect:*

To evaluate the behavioral distraction effect within each group, paired-samples *t*-tests were conducted to assess whether the distracting sounds influenced reaction time, accuracy, error rates, and miss rates. For between-group comparisons, we performed a two-way repeated-measures ANOVA with the factors: Group (4 levels) and Experimental Condition (distracting vs. nondistracting), followed by post hoc Scheffé tests to identify specific group differences.

*ERPs and Distraction Potential:*

Between-group differences in the amplitude and latency of obligatory ERP responses were assessed for: auditory N100, measured at the FCz electrode, and visual N1, measured at the Oz electrode. These differences were analyzed using one-way ANOVA followed by post hoc Scheffé tests.

For the *distraction potential*, grand-averaged difference waves (ERPs to distracting minus ERPs to nondistracting) were visually inspected in each group to identify the N1 enhancement, P3a, and RON components. Mean amplitudes and latencies for these components were extracted at the electrode showing the largest responses (FCz). Group differences in N1 enhancement, P3a, and RON were evaluated using one-way ANOVA with subsequent Scheffé post hoc tests, performed separately for amplitude and latency values.

Additionally, topographic maps for each component of the distraction potential were generated using the full set of 64 electrodes, referenced to the nose, to visualize scalp distribution patterns.

Spearman’s rank-order correlations were computed to assess associations between ERP components of the distraction potential and cognitive test scores.

Scores from the standard clinical cognitive evaluation were compared using one-wave ANOVA, followed by post hoc Scheffé tests.

## 3. Results

### 3.1. Standard Clinical Cognitive Evaluation

Healthy participants, across age groups, scored within the normal range on the MoCA, with scores of 28 for Y-group and 27 for O-group. In contrast, the patient groups had scores within the pathological range, with MCI patients scoring a mean of 25 and AD patients scoring a mean of 20 (*F*(1,20) = 16.3; *p* = 0.001). In the evaluation of attentional functions related to immediate recall (Craft Story), both patient groups recalled significantly fewer items compared to the healthy groups over 26 items; MCI: 19; AD: 6; (*F*(1,20) = 19.8; *p* = 0.002).

For attention assessed by the Trail Making Test, healthy participants completed the test faster than patient groups, with AD patients taking nearly twice as long as MCI patients (136 s vs. 74 s, respectively; *F*(1,20) = 5.7; *p* = 0.02). Memory evaluation results indicated that AD patients had the lowest scores on both verbal (Craft Story delayed recall) and visual memory tasks (Benson Figure delayed recall) compared to MCI patients (*F*(1,20) = 41.9; *p* = 0.001 for Craft Story delayed; *F*(1,20) = 14.0; *p* = 0.001 for Benson Figure delayed). All scores, including the normalized *z*-scores (UDS-3) are presented in Table 1.

### 3.2. Indices of Behavioral and Neurophysiological Distraction

**Figure 2:**
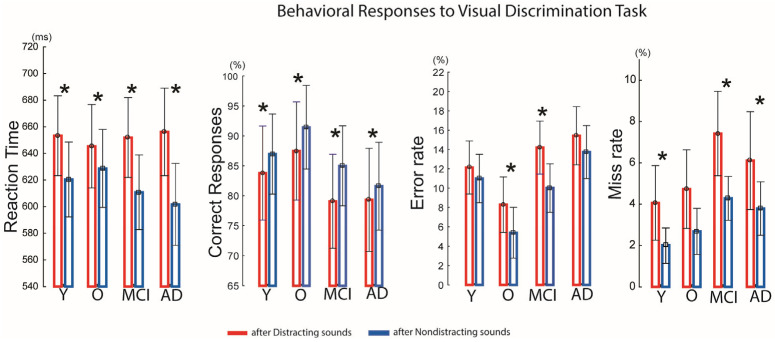
Behavioral distraction effects across groups. Graphics show results of behavioral responses to visual tasks preceded by auditory distracting and non-distracting sounds for each participant group. Reaction time, error rate, miss rate and correct response rate outcomes were calculated for both distracting and nondistracting sound conditions. The error rate is defined as the percentage of incorrect responses over the total number of trials. Miss rate is defined as the percentage of no response (within the allowed response time window = 1000 ms) over the total number of trials. Correct response rate is defined as the percentage of correct responses over the total number of trials. The asterisk (*) indicates statistically significant differences (*p* < 0.05). The vertical bars denote +/− standard errors.

The auditory–visual distraction paradigm was effective in each group: distracting sounds presented prior to visual stimuli significantly prolonged reaction times (RTs) in all groups compared with RTs following non-distracting sounds (Y: *t*(33) = 4.92, *p* < 0.01; O: *t*(16) = 2.50, *p* < 0.03; MCI: *t*(41) = 8.53, *p* < 0.01; AD: *t*(54) = 5.50, *p* < 0.01) (Figure 2).

Accuracy was also reduced by distracting sounds. In the younger group, correct responses decreased from 87% to 83% (*t*(−3.18) = −3.80, *p* < 0.02); in the older group, accuracy dropped from 91% to 87% (*t*(−3.60) = −2.82, *p* < 0.05); in the MCI group, from 85% to 79% (*t*(−5.91) = −4.16, *p* < 0.001); and in the AD group, from 81% to 79% (*t*(−2.87) = −2.14, *p* < 0.05).

Error rates following distracting sounds increased significantly in the older group (8.3% vs. 5.3%; *t*(2.9) = 2.47, *p* < 0.03) and the MCI group (14% vs. 10%; *t*(4.1) = 3.44, *p* < 0.04). Missed responses were also higher after distracting sounds in the younger group (4% vs. 2%; *t*(2.03) = 2.43, *p* < 0.03), the MCI group (7.5% vs. 4.2%; *t*(3.1) = 2.54, *p* < 0.02), and the AD group (6% vs. 3.5%; *t*(2.21) = 2.46, *p* < 0.05). For details, see Figure 2. However, between-group comparisons revealed no significant main effect of Group and no significant Group × Condition interaction, indicating that the reaction time slowing following distracting sounds, accuracy, error- miss-rates did not differ across groups, and that all groups were similarly behaviorally affected by auditory distraction.

### 3.3. Event-Related Brain Potentials

**Figure 3:**
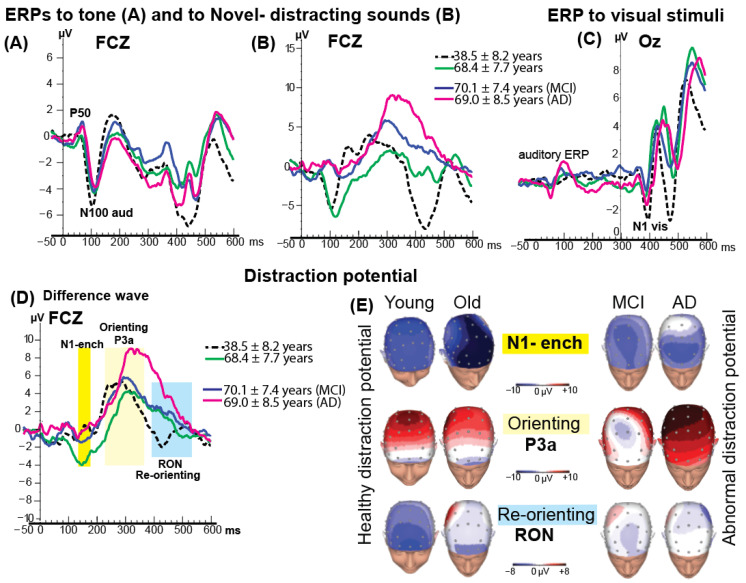
Event-related potentials (ERPs) and distraction potential across groups. (**A**) ERPs elicited by standard tone stimuli and (**B**) novel sounds recorded at the FCz electrode for each group. (**C**) ERPs to visual stimuli recorded at the Oz electrode for each group. (**D**) Grand-average distraction potential waveforms computed by subtracting tone-ERPs from novel-ERPs for each group. The distraction potential exhibited three well-formed phases, highlighted for each group. (**E**) Topographic scalp distribution maps for each phase of the distraction potential across groups. In the ERP waveforms, negative polarity is plotted downward. In the topographic maps, negativity is represented in blue and positivity in red, demonstrating average voltage distributions across all 64 electrodes (indicated by small circles) for each phase of the distraction potential: N1-enhancement, P3a, and reorienting negativity (RON).

An obligatory auditory N100 component, elicited by the tone at approximately 100 ms after sound onset, was observed in all groups (Figure 3A,B). No significant group differences were found in N100 latency or amplitude. However, in two healthy groups (*M* = 38 yo and *M* = 68 yo) the amplitude of N100 to distracting sound was larger than N100 to simple tone.

The visual stimuli (randomly appearing letters and numbers) were presented at 300 ms after the onset of the auditory stimuli. The grand-average visual N1 response to visual stimuli following the tone is shown in Figure 3C. Statistical comparisons of N1 to visual stimuli amplitude and latency across groups revealed no significant differences.

### 3.4. ERP Distraction Potential

Figure 3D shows the distraction potential across the four groups at the FCz electrode. The first phase of the distraction potential, the N1-enhancement, was significantly larger in amplitude in the healthy older group (*M* = 68 years) compared with the other groups, *F*(3, 41) = 4.98, *p* < 0.04.

The subsequent P3a component—index of the orienting to the distracting event, representing the second phase of the distraction potential, was elicited in all four groups. ANOVA on P3a latency revealed a significant delay in the older and patient groups compared with the younger group (Means: 320 ms vs. 290 ms, respectively; MCI = 315 ms, AD = 355 ms), *F*(3, 41) = 3.02, *p* < 0.04. No significant differences in P3a latency were observed among the older, MCI, and AD groups.

As shown in Figure 3D, the largest P3a amplitude was observed in the AD group. Topographical scalp distribution mapping confirmed this enlargement of P3a activity in AD group (see Figure 3E for details). At FCz, the mean (±SD) P3a amplitude was 11.0 ± 3.0 µV in the AD group, compared with 8.0 ± 1.3 µV in the younger group, 6.0 ± 2.7 µV in the older group, and 7.0 ± 1.9 µV in the MCI group.

Following the P3a component, a fronto-central negative-polarity waveform occurring within the 420–580 ms window was identified as the RON component (see Figure 3D, highlighted in blue). The RON represents the final phase of the distraction potential and is neurophysiologically associated with reorienting attention from the distracting event back to the ongoing visual discrimination task.

The younger group exhibited a significantly earlier peak latency (≈450 ms) and larger amplitude (−4 µV) compared with the older healthy group (540 ms/−2 µV), the MCI group (548 ms/−0.4 µV), and the AD group (553 ms/+0.3 µV). ANOVA revealed a robust main effect of group for RON latency, *F*(3,41) = 28.26, *p* < 0.001, and a significant effect for RON amplitude, *F*(3,41) = 3.50, *p* < 0.02.

A marked reduction in RON amplitude was noted in the AD group, evident at frontal electrodes. Topographical scalp distribution mapping confirmed this attenuation of RON activity (Figure 3E).

### 3.5. Correlation Analyses

**Figure 4:**
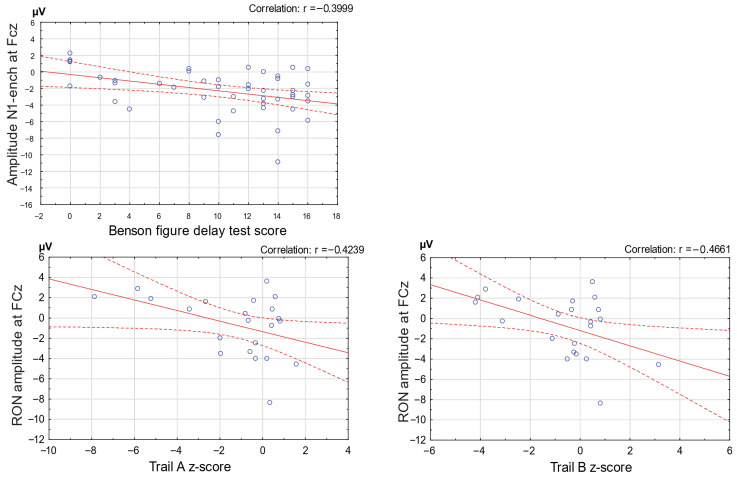
Correlations between distraction potential components and cognitive performance. The top panel shows the correlation between the mean amplitude of the N1-enhancement phase of the distraction potential and performance on the Benson Figure Delay Test (BFDT), a standard clinical measure of memory. Higher BFDT scores were associated with greater (more negative) N1-enhancement amplitudes. The bottom two panels depict correlations between executive function, assessed with the Trail Making Test (TMT), and the mean amplitude of the reorienting negativity (RON) in the two patient groups combined. These results reveal that lower (more negative) *z*-scores on the TMT were associated with smaller RON amplitudes.

Significant correlations were observed between ERP components of the distraction potential and cognitive test performance. The N1-enhancement amplitude was negatively correlated with Benson Complex Figure Delay scores when all participants were combined (*r* = −0.39, *p* < 0.05), indicating that faster change detection was associated with better visuospatial recall. In addition, the RON amplitude showed a significant negative correlation with Trail Making Test Parts A and B performance in the MCI and AD groups (*r* = −0.46, *p* < 0.05), suggesting that weaker reorienting responses were linked to poorer attentional shifting and executive control in the combined patient group (Figure 4).

## 4. Discussion

This study identified age-related changes in auditory distraction potential under normal aging conditions and, importantly, revealed additional alterations of brain activity associated with distractibility in MCI and AD patients. These findings provide objective evidence for both normative and pathological aging trajectories in the processing of auditory distractors during a discriminative visual task. Similarly to prior work showing that distraction-related ERPs such as N1-enchancement, P3a, and RON are sensitive to aging effects [13,16,33]. Our results extend this knowledge by distinguishing normal aging patterns from disease-related alterations in MCI and AD [34,35].

Our auditory–visual distraction paradigm was designed to engage two opposing processes during task performance. The *top-down process* required participants to focus attention on the visual discrimination of letters versus numbers, while the *bottom-up process* was triggered by irrelevant auditory distractors that automatically captured attention and interfered with the visual task. This dynamic competition between voluntary and involuntary attention was evident in both ERP responses and behavioral performance measures. Attention, as the brain’s mechanism for navigating environments filled with competing relevant and non-relevant stimuli, relies on the balance of these processes [36,37]. Importantly, both top-down and bottom-up brain mechanisms follow developmental and aging trajectories, making them highly relevant for understanding cognitive changes in general and auditory distraction specifically across the lifespan. In the present study, all groups, regardless of age or clinical condition, successfully processed auditory distractors, as reflected by the elicitation of the auditory N100 ERP, and reliably detected visual stimuli, as indicated by the visual N1 ERP. Behaviorally, participants across all groups performed the visual discrimination task at an acceptable level, achieving greater than 75% accuracy in correct responses. Importantly, auditory distractors efficiently captured attention away from the visual task as evidenced by significantly prolonged reaction times (RTs) and decreased accuracy following distracting sounds compared with non-distracting sounds across all groups, thereby confirming the presence of a distraction effect based on Tecce’s criteria [1].

The electrophysiological index of distraction is a distraction potential. These ERPs consist of three phases and functionally have been explained as follows: N1-enhancement—change detection between frequent sounds and unexpected novel sounds; P3a—involuntary orienting of attention to the novel sounds; and the RON—re-orienting attention back to the ongoing visual task [12,33,38]. From prior studies on aging, it is well established that healthy older adults show an increase N1 enhancement [39,40], but a reduction in P3–novelty activity compared with younger adults [41,42,43]. In our study, we found a similar pattern: the healthy older group showed an enlarged first phase of the distraction potential but exhibited smaller P3a amplitudes than the healthy young group (8 µV vs. 6 µV, respectively). Younger participants also demonstrated earlier P3a latencies compared with older adults (290 ms vs. 320 ms, respectively).

A novel finding we observed was in the AD group which showed no latency differences in the P3a compared with older healthy group, but exhibited abnormally larger P3a amplitude as compared to healthy same age group. This exaggerated orienting response to novelty reflects altered bottom-up attentional mechanisms in patients with AD. Anatomically, bottom-up mechanism has been linked to the inferior and middle frontal gyri and the right temporoparietal junction [44,45]. Our results suggest that AD pathology may selectively affect these cortical regions, distinguishing it from MCI pathology, which did not show the same pattern. This may indicate compensatory recruitment of frontal–temporo-parietal networks in AD, rather than the typical age-related decline observed in healthy aging. Topographic scalp distribution mapping further highlighted the difference between the MCI and AD groups in the P3a ERP response. This observation underscores the need for further detailed investigation across a broader range of cognitive impairments associated with pathological aging, in order to determine whether this pattern is specific to AD or represents a more general dementia-related marker of neurodegenerative processes. MEG evoked field potential studies have shown that the P3a involves a widely distributed network including prefrontal, cingulate, temporoparietal, and hippocampal regions [46]. Thus, by utilizing the distraction potential as a probe, future studies may be able to pinpoint the specific brain regions functionally impacted by AD versus by MCI.

The final phase of the distraction potential is the RON component, which is associated with the reorientation of attention back to the visual task following a distracting auditory event [14,43]. In our study, RON amplitude was reduced in healthy older participants compared with the younger group. This finding is consistent with previous findings showing age-related alteration in RON activity, interpreted as evidence of deterioration of attentional control with aging [33,42,47]. However, a more recent MEG evoked-field study reported the opposite pattern, with increased RON activity in older relative to younger participants [48]. These mixed results suggest that older adults may exhibit heterogeneous changes in the neuronal generators underlying the RON component. Such heterogeneity—even within the range of normal aging—may contribute to the divergent findings reported across studies.

In contrast, our patient groups with MCI and AD showed further attenuation and delayed latency of the RON response, suggesting that pathological aging leads to more consistent and progressive impairments in the reorienting of attention. Mechanistically, the RON has been linked to frontal and parietal control networks involved in shifting attention and executive reallocation of resources [43]. Thus, the reduced RON observed in MCI and AD likely reflects dysfunction in these networks, aligning with broader evidence of frontoparietal impairment in neurodegenerative disease.

In our study, we found that N1-enhancement and RON components of the distraction potential correlated with standardized cognitive *z*-scores on tasks assessing executive functions, including the Benson Complex Figure Delay, Craft Story Recall, and Trail Making Test. For studies like ours, which aim to identify potential brain indices—markers that are sensitive and specific to cognitive impairments associated with age-related neurodegeneration—this represents an important finding. However, larger studies with expanded samples are needed to determine the specificity and sensitivity of the distraction potential as a biomarker of cognitive impairment in the domain of distractibility.

There is a growing need for accessible and reliable auditory assessment tools that can be implemented in memory clinics to evaluate people with MCI and dementia. Our results suggest that between-group differences emerge most clearly in the later ERP components (P3a and RON), which index higher-order attentional control and reorienting. These differences highlight distinct pathophysiological changes in MCI versus AD and suggest that P3a and RON ERPs may be useful electrophysiological markers for differentiating between these conditions. Clinically, the auditory–visual distraction paradigm provides a sensitive framework for detecting early deviations from normative aging trajectories, offering potential value for identifying subtle impairments in individuals at risk for MCI and AD, who commonly experience increased distractibility. In addition, performance on this paradigm may help predict real-world functional difficulties, including compromised driving ability, reduced orientation in noisy environments, impaired multitasking, and diminished everyday behavioral efficiency—activities that are particularly vulnerable to deficits in attentional control and cross-modal inhibition.

## 5. Conclusions

The implications of our findings are both important and practical. First, they are relevant for clinical evaluation and diagnosis in older adults, offering electrophysiological markers of cognitive decline in involuntary switches in attention. Second, they provide insight into healthy aging by illustrating how the brain’s mechanisms for managing distraction—the distraction potential—evolve from young to older age. Finally, they highlight the utility of distraction potential ERP measures for research aimed at developing early biomarkers capable of distinguishing normal cognitive aging from cognitive impairments within the selective attention system.

## Figures and Tables

**Table 1:** Demographic and clinical cognitive evaluation results for healthy (young and old) and patients (MCI and AD) groups.

Characteristics	Young *n* = 12	Old n = 11	MCI n = 12	AD n = 10	*p*-Value
Age, mean SD years	38.5 [8.3]	70.9 [6.6]	70.7 [7.4]	69.0 [8.5]	O, MCI, AD n.s.
Female, n (%)	6 (50%)	6 (55%)	5 (45%)	5 (50%)	n.s.
Craft Story (immediate)	30.5 [1.5]	26.3 [6.9]	19.0 [6.5]	6.6 [6.5]	O > MCI > AD *p* = 0.01
UDS-3 *z*-score (Craft story)	NA	0.3 [0.9]	−0.3 [1.2]	−2.8 [0.4]	
Benson figure (immediate)	13.9 [2.3]	12.3 [2.4]	9.8 [4.0]	2.9 [4.3]	YO n.s. > MCI > AD *p* = 0.01
UDS-3 *z*-score (Benson figure)	NA	0.3 [0/9]	−0.5 [1.3]	−2.9 [1.4]	
Trail making test (Digits [s])	23.0 [5.8]	29.7 [9.3]	35.2 [19.9]	51.1 [27.6]	Y, O < AD *p* = 0.002
UDS-3 *z*-score (Trail (digits))	NA	0.1 [0.6]	−0.5 [2.0]	−2.0 [2.6]	
Trail making test (Digits and Letters [s])	47.3 [23]	58.4 [21]	74.6 [48]	136.4 [72.5]	Y, O < MCI, AD *p* = 0.02
UDS-3 *z*-score (Trail (Digits and Letters))	NA	0.6 [0.5]	0.08 [1.5]	−1.4 [1.8]	
Craft Story (delayed)	29.0 [8.2]	23.0 [5.8]	16.5 [7.2]	1.3 [1.9]	Y, O, MCI > AD *p* = 0.001
Benson figure (delayed)	13.9 [2.3]	12.6 [2.3]	9.6 [3.9]	2.9 [4.4]	Y, O, MCI, AD *p* = 0.001
Digit Span test (forward)	11.3 [1.7]	11.1 [2.0]	9.7 [2.1]	9.7 [2.0]	n.s.
Digit Span test (backward)	6.9 [2.7]	8.3 [2.3]	5.7 [2.1]	5.6 [2.5]	n.s.
MoCA	28.7 [1.5]	27.5 [1.6]	24.8 [2.2]	19.8 [3.6]	Y, O, MCI > AD *p* = 0.04
Epworth Sleepiness Scale (ESS)	6.0 [2.5]	4.0 [2.5]	8.0 [4.5]	5.9 [3.0]	Y vs. O n.s.; MCI vs. AD n.s.
Insomnia Severity Scale (ISI)	5.3 [3.7]	7.0 [3.0]	7.3 [3.6]	9.0 [5.4]	Y vs. O n.s.; MCI vs. AD n.s.

n.s. = not significant. NA = not applicable.

## Data Availability

The study data is available upon reasonable request to the corresponding author. The data are not publicly available due to privacy concerns but is available for personal request.

## Abbreviations

The following abbreviations are used in this manuscript:

ERPs	Event-Related Potentials
MEG	Magnetoencephalography
RON	Reorienting negativity
RTs	Reaction times
AD	Alzheimer’s Disease
MCI	Mild cognitive impairment
UDSv3	Uniform Data Set v3
MINT	Multilingual Naming Test
FAQ	Functional Activity Questionnaire
EEG	Electroencephalography
